# 496. Comparison of Severe COVID 19 and Influenza Infections in Pediatric Patients Requiring PICU in Bogota, Colombia

**DOI:** 10.1093/ofid/ofab466.695

**Published:** 2021-12-04

**Authors:** Ivan Felipe Gutiérrez Tobar, Tatiana Patiño, Paola Rico, Nicolas Figueroa, Juan Pablo Londono-Ruiz, Diana N Avila, Mayra A Sanabria, Angie Vergara, Paula Gonzalez, Carolina Rodriguez, Sandra Beltran

**Affiliations:** 1 Clinica Infantil Colsubsidio, Clínica Infantil Santa María del Lago, Bogotá, Distrito Capital de Bogota, Colombia; 2 Clínica Infantil Santa María del Lago, Bogota, Distrito Capital de Bogota, Colombia; 3 Clínica Pediátrica, Bogotá, Distrito Capital de Bogota, Colombia; 4 Clínica Infantil Colsubsidio, Bogotá, Distrito Capital de Bogota, Colombia; 5 Universidad El Bosque, Bogotá, Distrito Capital de Bogota, Colombia; 6 Fundación Universitaria Sanitas, Bogotá, Distrito Capital de Bogota, Colombia; 7 Universidad del Rosario, Bogotá, Distrito Capital de Bogota, Colombia; 8 Clínica infantil Santa María del Lago, Bogota, Distrito Capital de Bogota, Colombia; 9 Clínicas Colsanitas, Bogotá, Distrito Capital de Bogota, Colombia

## Abstract

**Background:**

COVID 19 infection represents a global threat and now a frequent cause of hospitalization in pediatrics. COVID 19, as well as Influenza virus could have a severe course. There are few studies, and no local or regional information comparing severe disease between COVID 19 and Influenza virus in children.

**Methods:**

Confirmed COVID 19 between March 2020 to October 2021 and influenza infections from Jan-2017 to dec-2019 were included. Asymptomatic or ambulatory COVID 19 infections were excluded. The main objective was to compare clinical, laboratory and outcome characteristic of PICU admitted patients.

**Results:**

71 patients were included, 32(45,1%) with COVID 19 and 39 (54,9%) influenza virus. COVID 19 patients were older than influenza patients: 67 (20,5-143) vs. 10 (2- 46) p=0.0002. The majority of influenza patients were younger than two years, with different distributions in COVID 19 patients. Figure 1. Respiratory distress was more frequent in influenza (92,3% vs. 62,5%) p=0.002, but exanthema (28,1% vs 2,6%), shock (68,7% vs. 7,7%) and central nervous system manifestations (40,6% vs. 7,7%) were significantly more common in COVID19 than in Influenza respectively. COVID 19 had lower platelets and lymphocyte counts than inlfuenza. There were no differences in treatment, nor deceased either, but Influenza patients had slightly longer hospital stays 12 (7 – 23) vs. 9.5 (6–15.5) p=0.1592 than COVID 19 (Table 1).

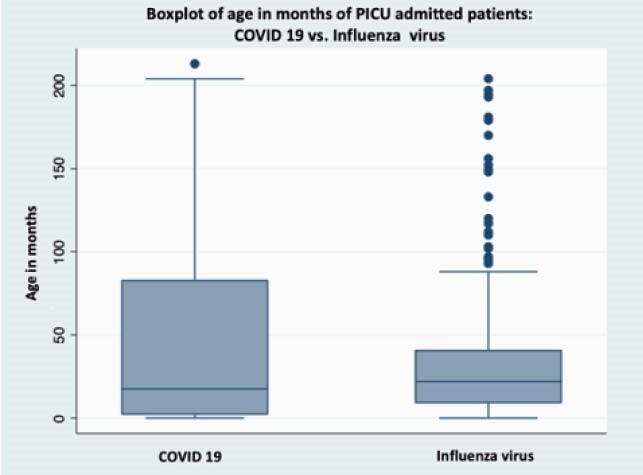

Table 1. Comparison of demographic and clinical characteristics of COVID 19 vs. Influenza patients.

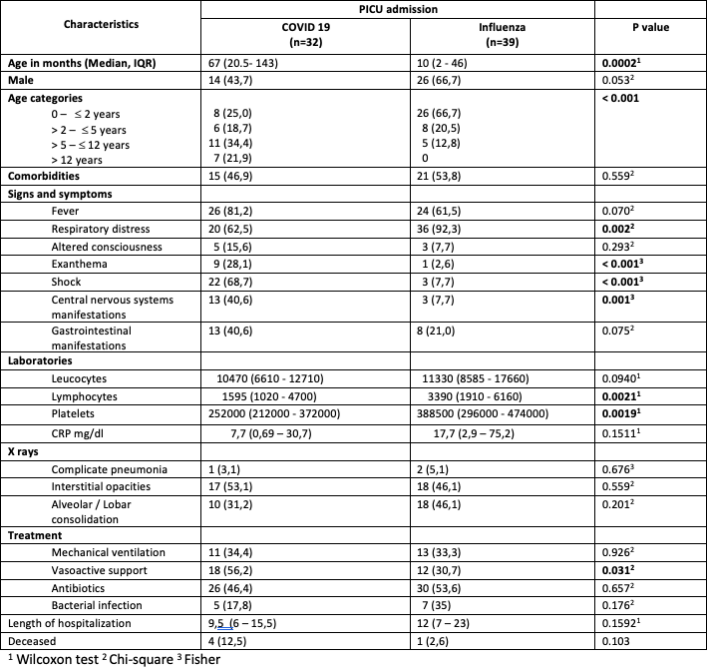

**Conclusion:**

COVID 19 and influenza severe infections can have some differences including age of presentation. Inlfuenza main manifestation requiring UCIP is respiratory distress, while COVID19 can have other presentations including shock and central nervous manifestation. Lower lymphocyte counts as well as lower platelets were significantly more common in COVID 19 patients. Although there are no unique characteristics of each infection, some particularities could guide clinician to the etiology of the infection.

**Disclosures:**

**Ivan Felipe Gutiérrez Tobar, n/a**, **Pfizer and MSD** (Advisor or Review Panel member, Research Grant or Support, Speaker’s Bureau, Has received support from Pfizer and MSD for participation in congresses and has received conference payments from Pfizer)**Pfizer and MSD** (Speaker’s Bureau, Other Financial or Material Support, Has received support from Pfizer for participation in congresses) **Sandra Beltran, n/a**, **Pfizer** (Other Financial or Material Support, Has received support from Pfizer for participation in congresses)

